# Physics-Driven Construction of Compact Primitive Gaussian Density Fitting Basis Sets

**DOI:** 10.1021/acs.jctc.5c01219

**Published:** 2025-10-25

**Authors:** Kshitijkumar A. Surjuse, Edward F. Valeev

**Affiliations:** Department of Chemistry, 1757 Virginia Tech, Blacksburg, Virginia 24061, United States

## Abstract

We present a model-assisted density fitting (MADF) basis set generator, an algorithm for generating primitive atomic Gaussian density fitting (DF) basis sets (DFBSs) from a contracted Gaussian orbital basis set (OBS). The MADF algorithm produces DFBSs suitable for accurate robust DF approximation of 2-particle interactions in mean-field and correlated electronic structures. The algorithm is designed to (a) saturate the OBS product space by a large regularized set of primitive solid-harmonic Gaussian shells with nonuniform distribution of exponents, followed by (b) pruning of the shells according to their contributions to the 2-body energy of a correlated atomic ensemble. Building the DFBS generator model almost exclusively on mathematical and physical principles allows one to limit the number of parameters that control the density fitting error to three, with a single set of parameters sufficient for computations with all basis cardinal numbers, with and without correlation of core electrons, with and without scalar and spin-dependent relativistic effects, spanning almost all of the Periodic Table. Performance assessment included basis sets up to quadruple-ζ quality from several major basis set families, using molecules composed of main-group, d-block, and f-block elements. The resulting DF errors in Hartree–Fock and second-order MP2 energies (with relativistic all-electron treatments, when appropriate) were on the order of 20 and 10 μ*E*_h_ per electron, respectively.

## Graphical abstract

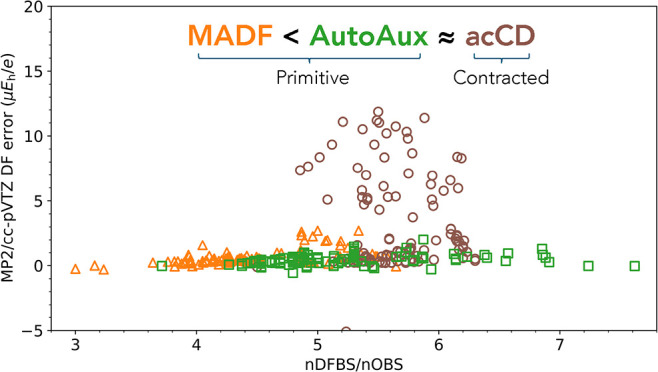

## 1 Introduction

Density fitting (DF),^1–6^ also known as resolution of identity (RI),^7,8^ is a widely used cost and complexity reduction technique for approximate evaluation of various operators in the electronic structure theory, most commonly the electron repulsion integrals (ERIs). The cost of ERI evaluation and their storage are often crucial limitations in practical simulations of electronic structure. The factorization of ERIs using the DF approximation can be exploited in various ways to gain significant speedups and reduce storage requirements,^9–16^ such as to reduce the cost of electrostatic potential evaluation^10,17^ and to facilitate correlated and relativistic computations.^17–21^ DF has also recently emerged as the route to deeper factorizations of the ERI tensor such as real-space tensor hypercontraction,^22^ algebraic pseudospectral,^23^ canonical polyadic,^24^ and interpolative separable density fitting (ISDF).^25^ Although our sole focus in this work is on the Gaussian AOs, note that density fitting is a key enabling technology for other choices of AOs such as Slater-type^26^ and numerical AOs.^27^

In the DF approach, the AO products (or “densities”) in the ERIs are expanded in a special-purpose density-fitting (or auxiliary) basis of AOs. Traditionally, density-fitting basis sets (DFBSs) are manually optimized for each orbital basis set (OBS) in ad hoc manner.^17,28–37^ For efficiency reasons not only are DFBS matched to the OBS but they are also matched to specific use cases; e.g., DFBS for Coulomb fitting (RI-J),^29,31^ Coulomb and exchange fitting (RI-JK)^17,32^ or for the second-order Mo̷ller-Plesset (MP2) correlation energy (RI-C).^17,28,30,33,34,36,37^ DFBS optimization is usually done with cumbersome iterative HF and MP2 calculations on a training set of atoms and molecules.

Due to the significant effort involved in the DFBS development, there are only a few OBS families for which there are matching DFBS, and those often only focus on the top half of the Periodic Table. Among the commonly used basis sets, only the correlation consistent basis sets and the def2- (TURBOMOLE) basis sets have matching DFBS. Even for these families there are significant coverage gaps; e.g., correlation-consistent OBSs such as cc-pVXZ, cc-pCVXZ, and cc-pwCVXZ^38–40^ have DFBS coverage gaps in the first to third rows of the Periodic Table.^41^ Only the def2- family has a thorough DFBS coverage for atoms up to Rn (with atoms heavier than Kr using effective core potential).^17,31,41–44^ Unfortunately, for heavier elements, there are no useful DFBS coverage. For example, OBS families such as ANO-RCC basis sets,^45–47^ cc-pwCVXZ-DK/-DK3/-X2C^48–51^ basis sets, the x2c-XZVP^52,53^ family of basis sets, and Dyall basis sets^54–56^ do not have the corresponding DFBS designed for correlation calculations. The only two relativistic DFBSs available in Basis set exchange (BSE)^41^ database are x2c-JFIT and x2c-universal-JFIT,^53^ but they are designed only for Coulomb fitting and are not suitable for many-body correlated simulations or even for hybrid Kohn–Sham DFT computations.

To address the challenges of ad hoc optimization of DFBS many black-box methods have been proposed for generating (rather than optimizing) DFBS for a given OBS have been proposed.^57–63^ A DFBS generator can be viewed as an OBS → DFBS map controlled by zero or more model parameters. The key difference between DFBS generators and manual optimization is that no nonlinear optimization is involved in the definition of the map. A map can be defined straightforwardly to produce exact atomic DFBS (=DFBS that is exact for any computation on a single atom), but such DFBS is too large to be practical. Nevertheless, such an exact map is typically the starting point for the design of approximate DFBS generators. Nearly all DFBS generators in practice are approximate, i.e. they produce DFBS that are not exact even for a single atom.

Most DBFS generators are designed only for DF in the context of evaluating the Coulomb potential of a charge density. These include the AutoABS method by Yang et al.,^57^ atomic Cholesky decomposition (aCD) and atomic compact Cholesky decomposition (acCD),^64,65^ long-range corrected auxiliary basis sets by Hellmann et al.,^58^ even tempered auxiliary basis sets with shared exponents by Daz-Tinoco et al.^59^ and a similar approach has also recently been reported for relativistic OBSs.^60^

DFBS generators designed for mean-field and correlated electronic structure simulation include the AutoAux procedure by Stoychev et al.,^61^ methods proposed by Lehtola,^62,63^ and PySCF built-in generator of even-tempered DFBSs.

The AutoAux method, available in the ORCA software package^66^ and in the command-line interface of Basis Set Exchange (BSE),^41^ produces a primitive even-tempered DFBS that can be applied universally (in mean-field and correlated simulations). The AutoAux generator uses even-tempered sequences of Gaussian primitive AOs to span the OBS-deduced range of exponents for each angular momentum block. The exponent ranges and even-tempered ratios vary with the angular momenta (*L*) and the atomic number *Z* (these even-tempering parameters are prescribed for up to *L* = 7; the BSE implementation of AutoAux uses the same exponent ratio for *L* ≥ 7). The generator also limits the highest angular momentum of DFBS AOs below what is needed for exact atomic DF, in a manner that depends on the highest angular momentum of the occupied orbitals. AutoAux as a result is quite complex, defined with more than a dozen model parameters in total.

PySCF’s simple built-in generator^67^ produces even-tempered primitive DFBS and is very similar in spirit to AutoAux. It has fewer model parameters (e.g., even-tempering ratio is not graded with angular momenta) and claims to offer similar accuracy to AutoAux, although no extensive comparison exists.

Lehtola presented a DFBS generator that produces nearly exact atomic DFBS composed of primitive solid-harmonic Gaussian (SHG) AOs.^62^ First, the OBS AOs on a given atom are used to generate a base pool of primitive SHGs by approximating each angular momentum channel of solid-harmonic OBS AO products by a single solid-harmonic AO. The resulting pool is then regularized by pivoted Cholesky decomposition (pCD) of the two-center two-electron Coulomb integral matrix, with the accuracy of the resulting primitive DFBS controlled robustly by a single threshold. Although the resulting DFBSs are nearly exact, they are extremely large, lead to high condition numbers in molecules, and contain primitive Gaussians with twice the angular momentum of the highest angular momentum (*L*_OBS_) of functions in the parent OBS. Hellmann and Neugebauer^68^ extended Lehtola’s method^62^ to include exact spherical functions with mixed angular momentum functions instead of creating multiple angular momentum channels; however, it does not address the large number of primitives in the generated DFBS. Lehtola addressed the latter issue in his recent work^63^ by extending the algorithm to prune high-angular momentum functions out of the DFBS with a tunable parameter. The algorithm then uses Kállay’s^69^ contraction method with singular value decomposition (SVD) to contract the DFBS, further reducing its size.

An attractive alternative to density fitting that avoids these issues and provides some of its advantages is the (pivoted) Cholesky decomposition (CD).^70^ Any positive-definite 2-particle interaction represented in an arbitrary OBS can be decomposed with accuracy controlled by a single threshold. CD has been used for electronic structure simulations in both relativistic^71–73^ and nonrelativistic^74–76^ contexts and close connections between CD and DF have been demonstrated.^77^ Although the accuracy of CD can be robustly pushed beyond the reach of conventional density fitting, the cost of molecular CD can be substantially higher than that of DF-based approaches and CD is not as universally applicable black-box technology as desired.^78^ The use of CD for construction of atomic density fitting basis sets, under the name of atomic CD (aCD), was pioneered by Aquilante et al.^64^ The improved version of aCD, termed atomic compact CD (acCD),^65^ was proposed later that greatly reduced the number of primitive and contracted DF AOs. Both the aCD and acCD approaches can be viewed as black-box 1-parameter DFBS generators and are also unbiased toward mean-field and correlated methods, albeit they produce deeply contracted DFBS AOs.

To navigate the severe gaps in the coverage by existing manually constructed DFBS and the notable shortcomings of the existing DFBS generators we attempted to design a new DFBS generator that produces (a) primitive Gaussian DFBSs, (b) of comparable size and accuracy as the manually optimized DFBS, (c) usable in the context of mean-field and correlated simulation, and (d) has as few adjustable parameters as possible to ensure maximum universality. By combining the existing ideas for spanning the product space of OBS AOs with new ideas for regularizing and pruning the exact DFBS using atomic correlated ensemble RDMs we arrived at a DFBS generator dubbed MADF. The purpose of this manuscript is to describe it and assess its performance. Following a quick recap of density fitting in Section 2.1 we describe the 2 key ingredients of the MADF generator: the regularization of the complete set of candidate primitives (described in Section 2.2) and subsequent pruning by a 2-body energy estimator model (described in Section 2.3). Section 3 describes the technical and details of the parameter training regiment. In Section 4 we document the performance of the DFBSs generated by our method against the manually optimized DFBSs and the DFBSs generated by the AutoAux algorithm and acCD.

## 2 Formalism

### 2.1 Density Fitting

DF approximates product of AOs (“density”) ϕ_μ_(**r**)­ϕ_ν_(**r**) ≡ (**r**|μν) as a linear combination of DFBS AOs ϕ_*X*_(**r**):

$$|\mu \nu )\approx \sum_{X}C_{\mu \nu }^{X}|X)\equiv \left|\tilde{\mu \nu }\right)$$ 1

The fitting coefficients *C*_μν_^*X*^ are obtained by minimizing a norm of the density error $|\delta_{\mu \nu })\equiv \left|\mu \nu \right)-\left|\tilde{\mu \nu }\right)$ . To minimize the error in the diagonal elements of the matrix representation of a positive operator *Ô*, namely in (μν|*Ô*|μν), the optimal choice of the error norm is the expectation value of operator *Ô* itself (as is done throughout this work):

$${∥f∥}_{Ô}\equiv (f|Ô|f)$$ 2

Such choice makes the error in (μν|*Ô*|ρσ) quadratic in the density fitting errors and is a special case of robust density fitting;^79,80^ if the fitting norm were defined with operator *Ŵ* ≠ *Ô* the explicit robust fitting approach would be necessary and variational extensions^81^ could be introduced for convenience. Least-squares minimization of ||δ_μν_||_*Ô*_ is equivalent to solving the following equation:

$$Ô|\mu \nu )=\sum_{Y}Ô|Y)C_{\mu \nu }^{Y}$$ 3

In global DF eq 3 is projected on every DF AO in the system to produce the usual system defining the fitting coefficients:

$$\left(X|Ô|\mu \nu \right)=\underset{Y}{\sum }\left(X|Ô|Y\right)C_{\mu \nu }^{Y}$$ 4

Clearly, DF is a misnomer (and so is RI), as in the most common scenario, what is being “fitted” (minimized) is the *L* ^2^ norm of *Ô* ^1/2^|δ_μν_), not of |δ_μν_) itself. For the most important case, where *Ô* is the Coulomb potential operator *Ĵ*,

$$\left(r|Ĵ|f)\equiv \int dr^{\prime }\;{\left|r-r^{\prime }\right|}^{-1}f(r^{\prime }\right)$$ 5

the robust fitting minimizes the *L* ^2^ norm of the fitting error in the electric field generated by the AO product.

DFBS generators usually^57–63,66^ work in a single-atom context, i.e., DFBS is constructed using information about products on OBS AOs on a single atom. It is reasonable to question whether this is sufficient. Assuming a system composed of a single atom type, global density fitting in a system of *n* atoms will be able to fit every such 1-center product of OBS AOs using *n* times as many DFBS AOs as in a single atom. Indeed, for long-range *Ô* the fitting of even strongly localized AO products generally involves DFBS AOs from far away.^82^ Thus, 1-center products in a molecule can borrow DFBS AOs from other centers to make the fitting more accurate than in a single atom. This suggests that a single atom poses a more challenging DF setting than a molecule, which supports the use of a single-atom setting for the generation of DFBS.

### 2.2 Products of Primitive Gaussians AOs

The density fitting becomes exact if the span of DFBS matches the span of the OBS AO products. Because DFBS AOs are atom-centered but the products of AOs in a molecule are generally centered between atoms, exact DF is only possible for a single atom. We then confine ourselves to a single atom. Furthermore, we will limit ourselves to the case of OBS and DFBS composed of solid-harmonic Gaussian (SHG) AOs. Unnormalized primitive SHG with exponent α is defined as^83,84^

$$\chi_{\alpha ,l,m}(r)=r^{l}\;\exp(-\alpha r)y_{lm}(\theta ,\phi )$$ 6

$$y_{lm}(\theta ,\phi )=(-1{)}^{m}P_{l}^{\left|m\right|}(\theta )\{\begin{array}{ll} \cos(m\phi ), & m\geq 0\\ \sin(\left|m\right|\phi ), & m<0 \end{array}$$ 7

Whereas product of any number of concentric primitive Cartesian Gaussians is a single Cartesian Gaussian (modulo normalization), product of concentric SHGs χ_α_μ_,*l*_μ_,*m*_μ__ and χ_α_ν_,*l*_ν_,*m*_ν__ is a linear combination of *r* ^*l*_μ_+*l*_ν_–*L*^χ_α_μ_+α_ν_,*L*,*m*_μ_+*m*_ν__ for max­(|*l*_μ_ – *l*_ν_|, *m*_μ_ + *m*_ν_) ≤ *L* ≤ *l*_μ_ + *l*_ν_. Clearly, the contribution to the product from the Clebsch–Gordan channel *L* < *l*_μ_ + *l*_ν_ is an SHG multiplied by *r* ^*l*_μ_+*l*_ν_–*L*^. Following Stoychev,^61^ Lehtola proposed to account for this effect by approximating each such contribution by a single SHG of angular momentum *L* with the following effective exponent (see Appendix II of ref 62):

$$\alpha_{\mathrm{eff}}^{L}={\left[\frac{\Gamma \left(l_{\mu }+l_{\nu }+\frac{3}{2}\right)\Gamma (L+2)}{\Gamma \left(L+\frac{3}{2}\right)\Gamma (l_{\mu }+l_{\nu }+2)}\right]}^{2}(\alpha_{\mu }+\alpha_{\nu })\{\begin{array}{ll} =\alpha_{\mu }+\alpha_{\nu }, & L=l_{\mu }+l_{\nu }\\ >\alpha_{\mu }+\alpha_{\nu }, & L<l_{\mu }+l_{\nu } \end{array}$$ 8

We follow this prescription here to define the “complete” set of candidate primitive SHGs for a given product of primitive SHG OBS AOs.

Let C denote the “complete” set of all unique solid harmonic primitive AOs generated thereby from all products of primitive OBS AOs on a given atom. The set C can be used as DFBS for nearly exact density fitting in an atom (a) for any contraction of the generating set of primitive OBS AOs and (b) for any operator. However, even in a single atom such DFBS is highly linearly dependent and overcomplete,^62,85^ therefore, it is unusable with finite precision arithmetic. Moreover, such a basis would be extremely large, defeating the purpose of the DF approximation. Hence, a compact DFBS is needed to represent the product space of AO functions. Since the set C is overcomplete for use as a DFBS for an atom, the unimportant and linearly dependent functions must be trimmed out of this set. Lehtola proposed to do such pruning by pCD.^62^ However, even after pCD the resulting DFBSs are still large, as illustrated in Figure 1. While pCD reduces the density of the complete set, it largely leaves its range of exponents intact (at least for lower *L*), and it still keeps many pairs of adjacent exponents with small ratios (≪2). The problem is especially pronounced for the high angular momentum (*L* = 6).

**Figure 1:**
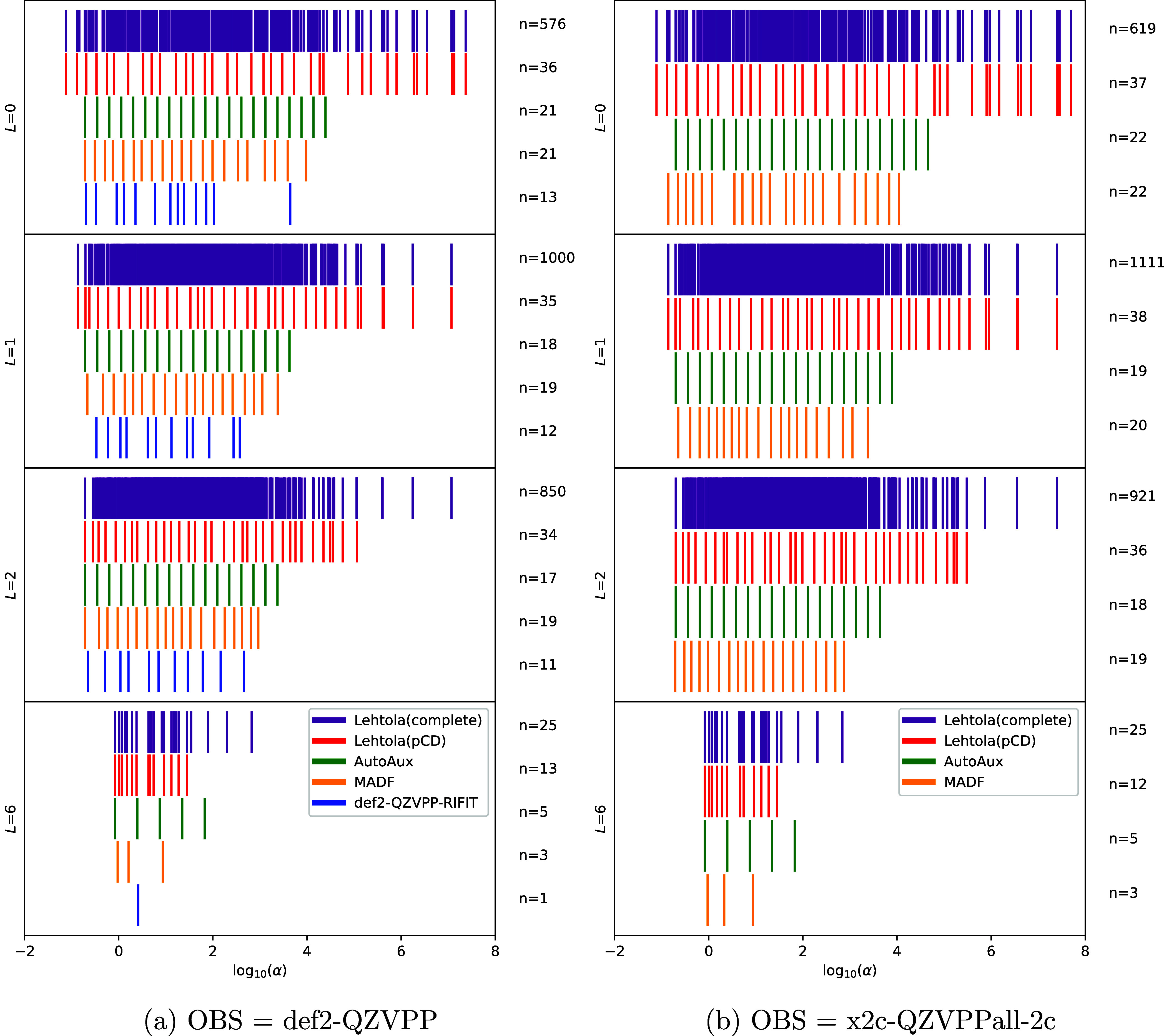
Exponents of DFBS generated (or manually optimized) for Kr atom with representative nonrelativistic and relativistic OBS using various methods scattered on a logarithmic number line for different angular momenta i.e., *L* = {0, 1, 2, 6}. The number of exponents is shown on the right of each scatter plot. Figure 1a shows distribution of DFBS exponents for nonrelativistic OBS def2-QZVPP and Figure 1b for relativistic OBS x2c-QZVPPall-2c. The plot depict Lehtola’s complete set (shown in purple), Lehtola’s pCD-regularized set (shown in red) obtained using ERKALE^86^ with pCD threshold 10^–7^ as recommended in ref 62, even-tempered exponents produced by AutoAux (shown in green) and set of exponents produced by the MADF algorithm (shown in orange). Distribution of exponents of manually optimized DFBS def2-QZVPP-RIFIT is also shown (in blue) in Figure 1a.

**Figure 2:**
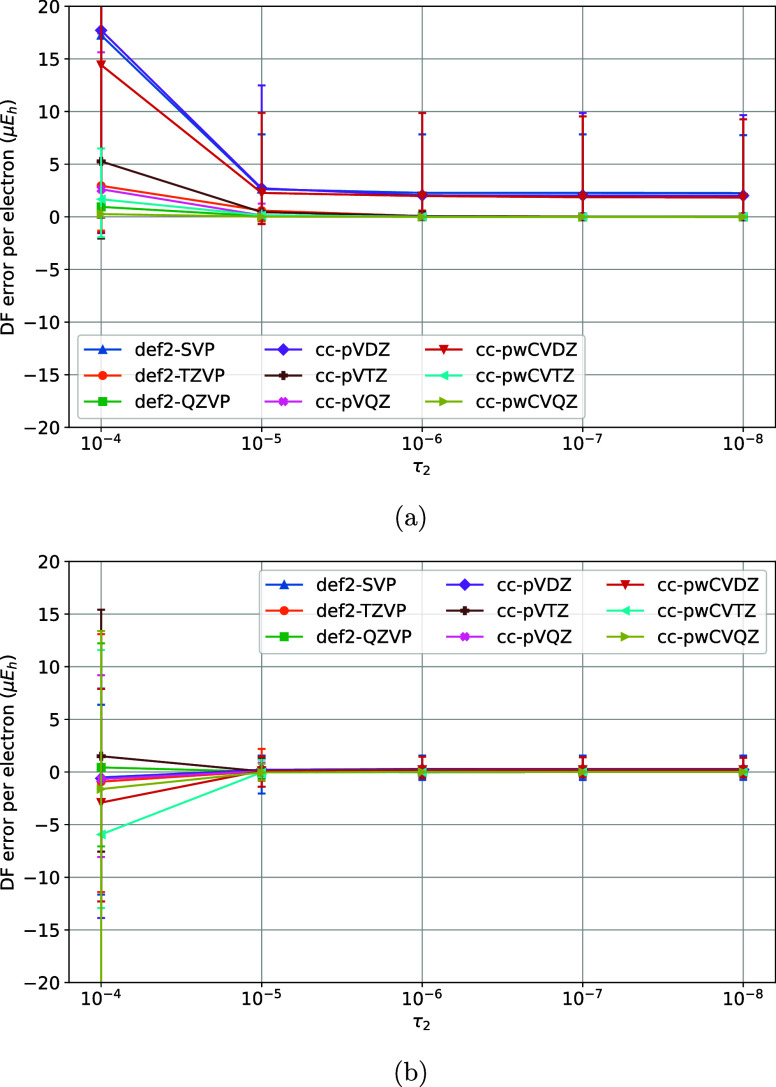
Variation of DF errors of nonrelativistic (a) HF and (b) MP2 energies of the TS1 training set vs the τ_2_ model parameter of the MADF generator.

**Figure 3:**
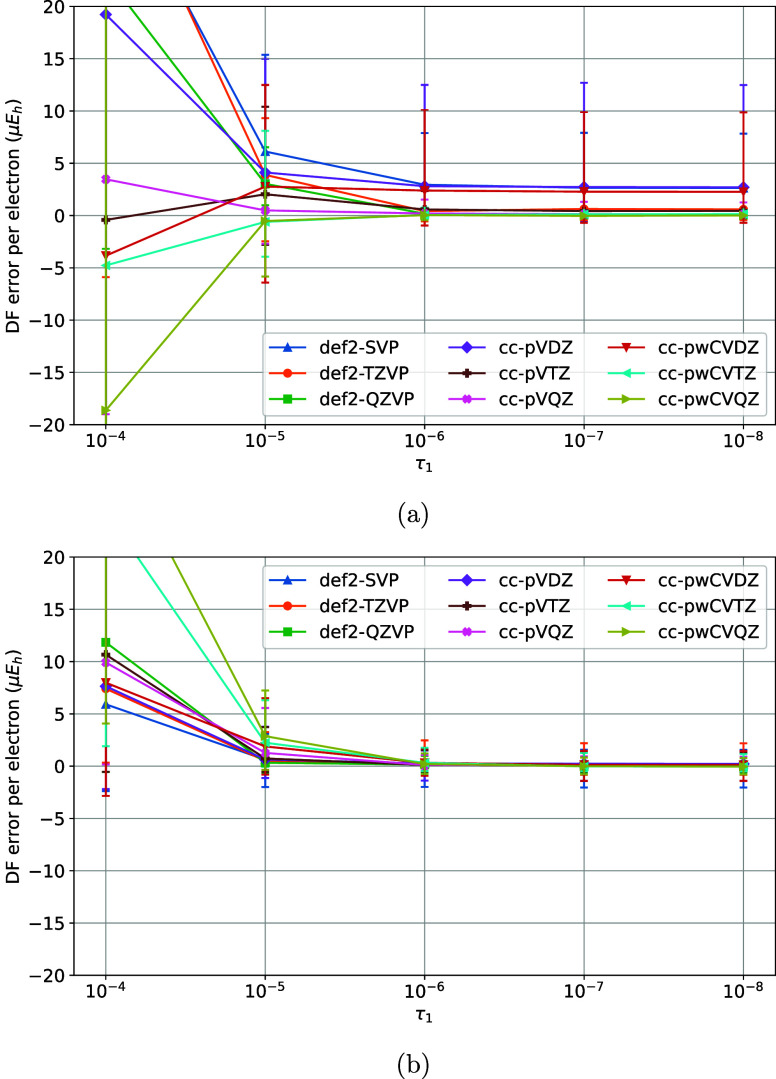
Variation of DF errors of nonrelativistic (a) HF and (b) MP2 energies of the TS1 training set vs the τ_1_ model parameter of the MADF generator.

**Figure 4:**
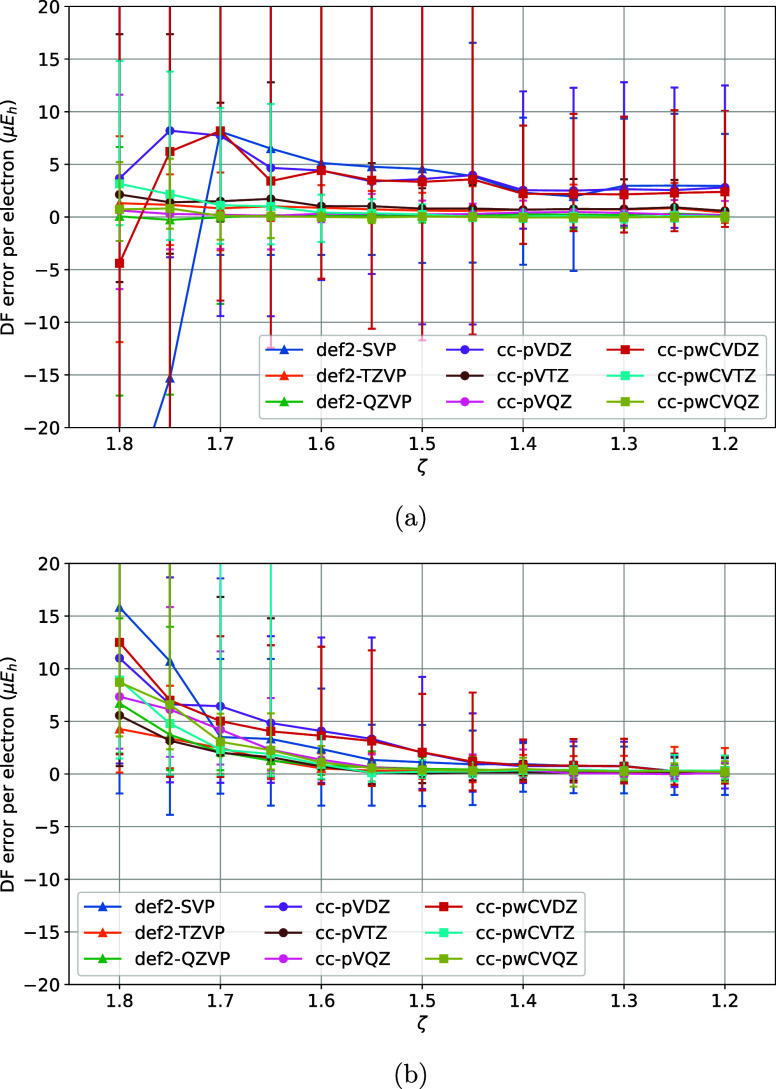
Variation of DF errors of nonrelativistic (a) HF and (b) MP2 energies of the TS1 training set vs the ζ model parameter of the MADF generator.

**Figure 5:**
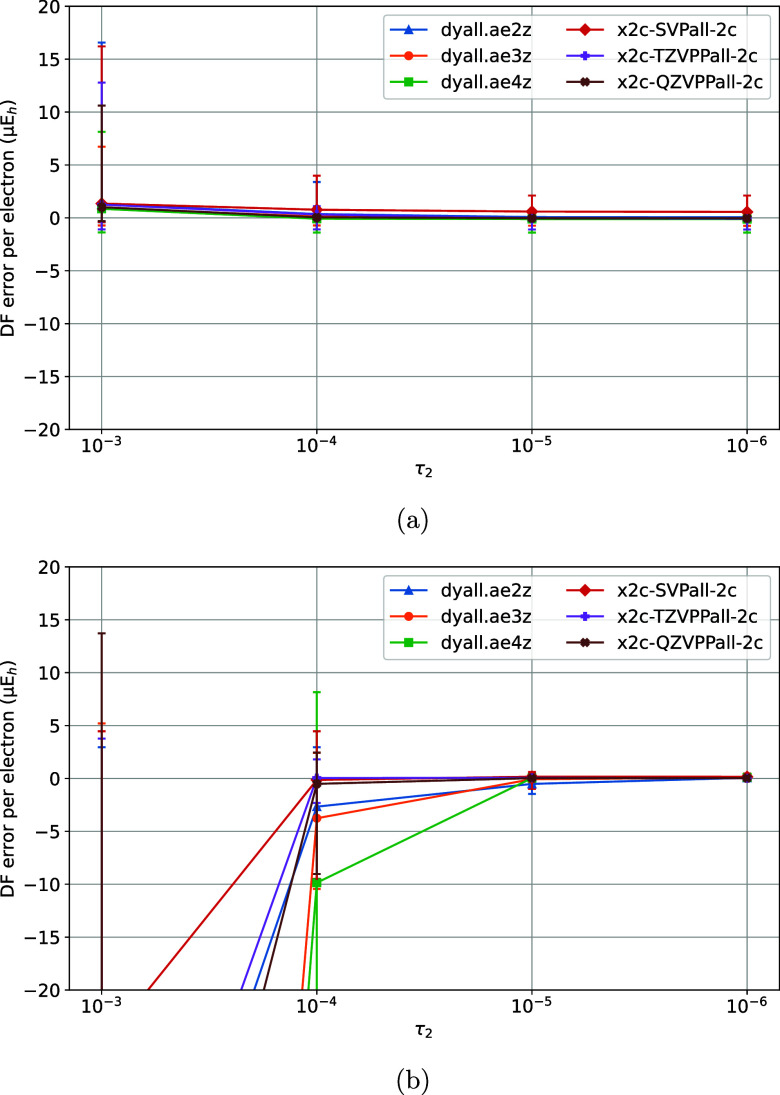
Variation of DF errors of (relativistic) (a) X2C-HF and (b) X2C-MP2 energies of the TS2 training set vs the τ_2_ model parameter of the MADF generator.

**Figure 6:**
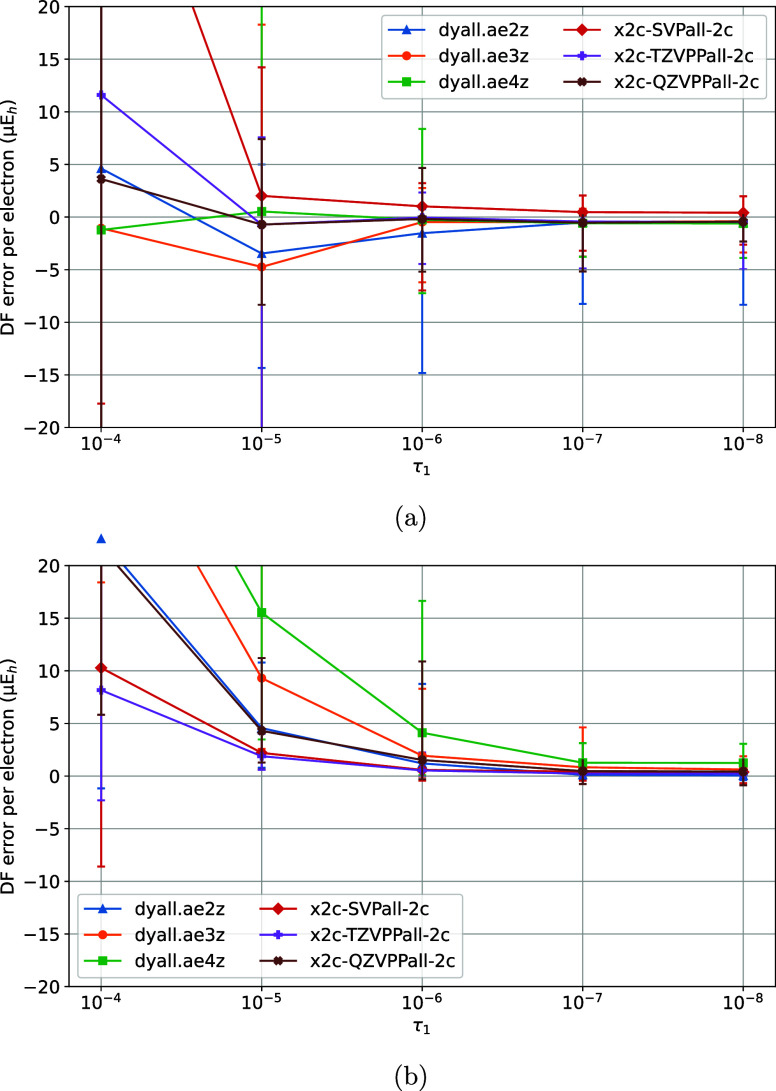
Variation of DF errors of (relativistic) (a) X2C-HF and (b) X2C-MP2 energies of the TS2 training set vs the τ_1_ model parameter of the MADF generator.

**Figure 7:**
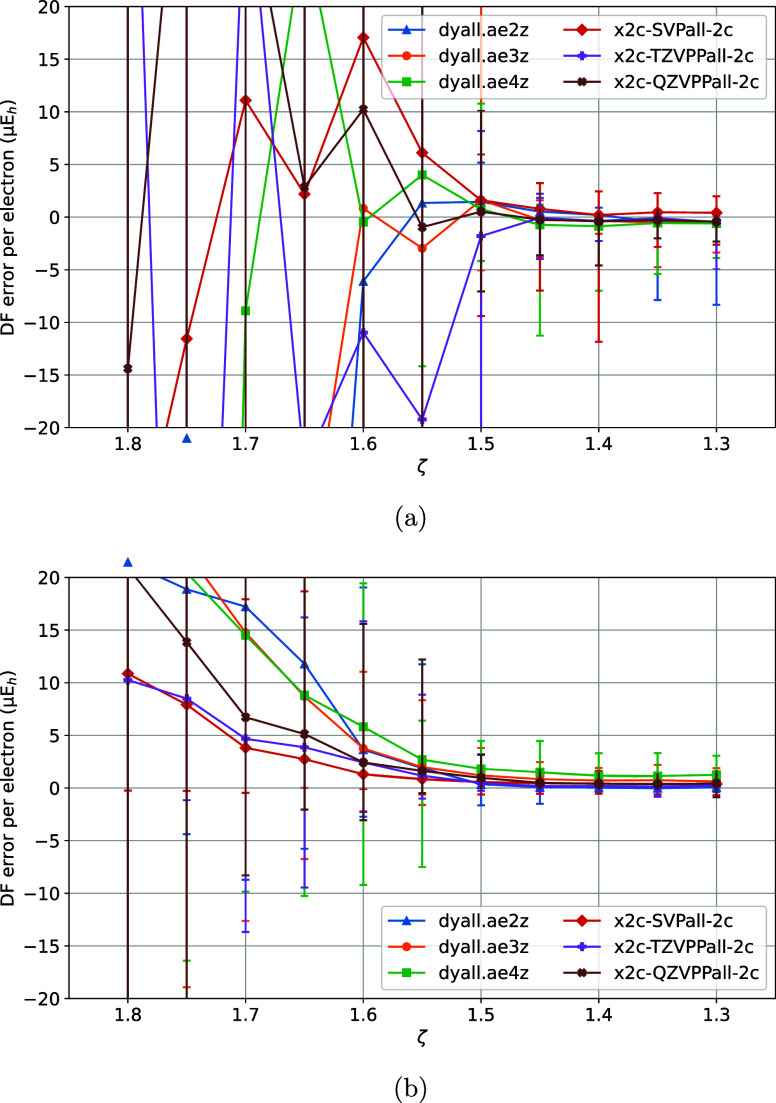
Variation of DF errors of (relativistic) (a) X2C-HF and (b) X2C-MP2 energies of the TS2 training set vs the ζ model parameter of the MADF generator.

**Figure 8:**
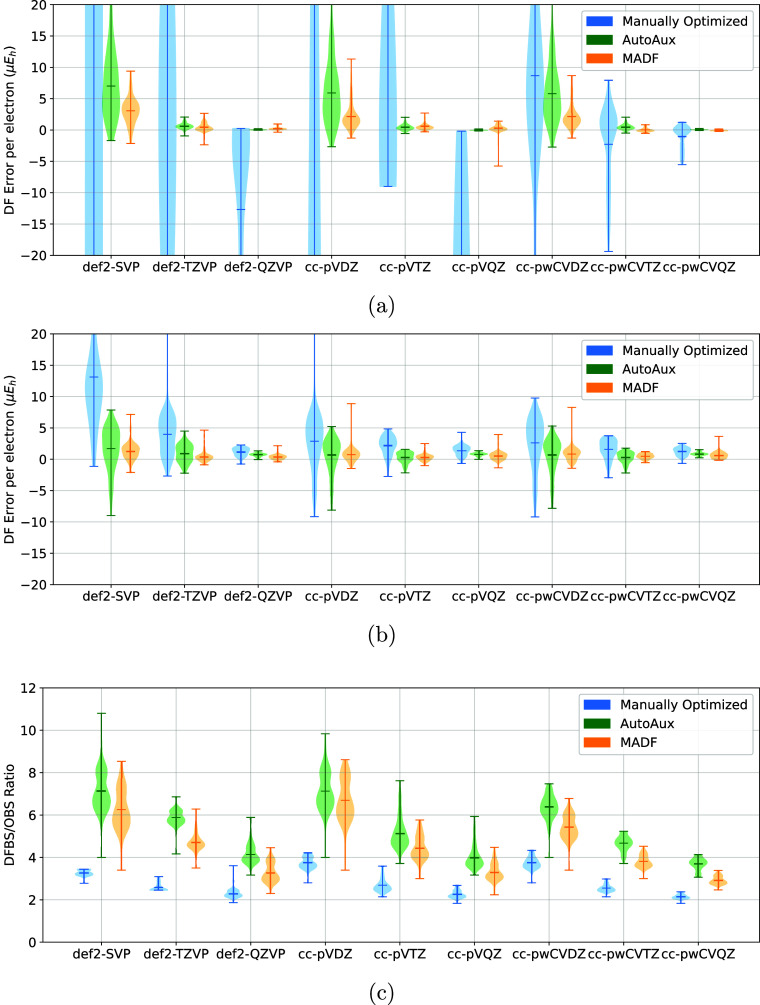
Comparison of DF errors of (a) HF and (b) MP2 energies of the G2 test set obtained with manually optimized, AutoAux, and MADF DFBSs. (c) Comparison of DFBS sizes, relative to that of the corresponding OBS.

**Figure 9:**
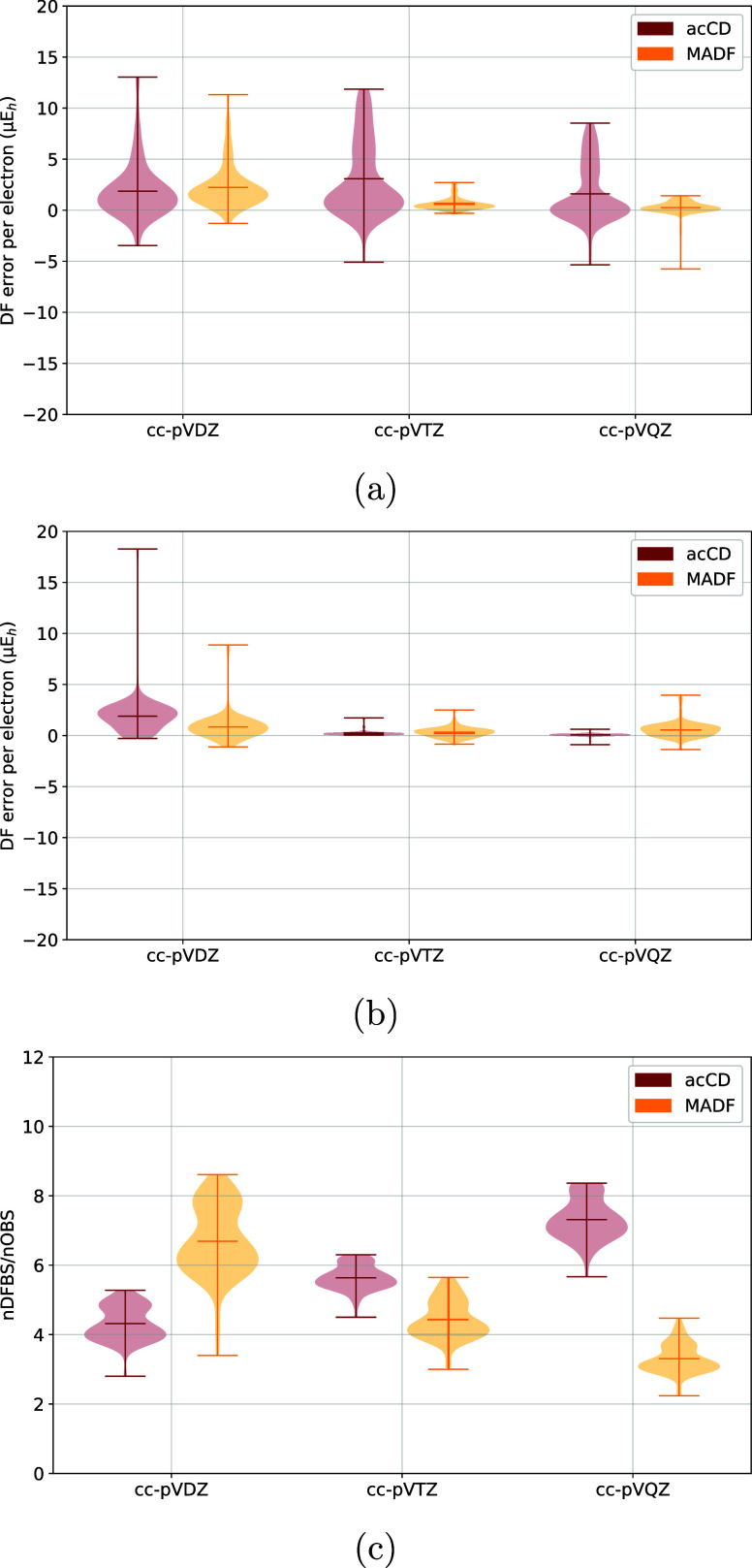
Comparison of DF errors of (a) HF and (b) MP2 energies of the closed-shell subset of the G2 test set obtained with acCD and MADF DFBSs. (c) Comparison of DFBS sizes, relative to that of the corresponding OBS. For acCD, the number of contracted DF AOs was used.

**Figure 10:**
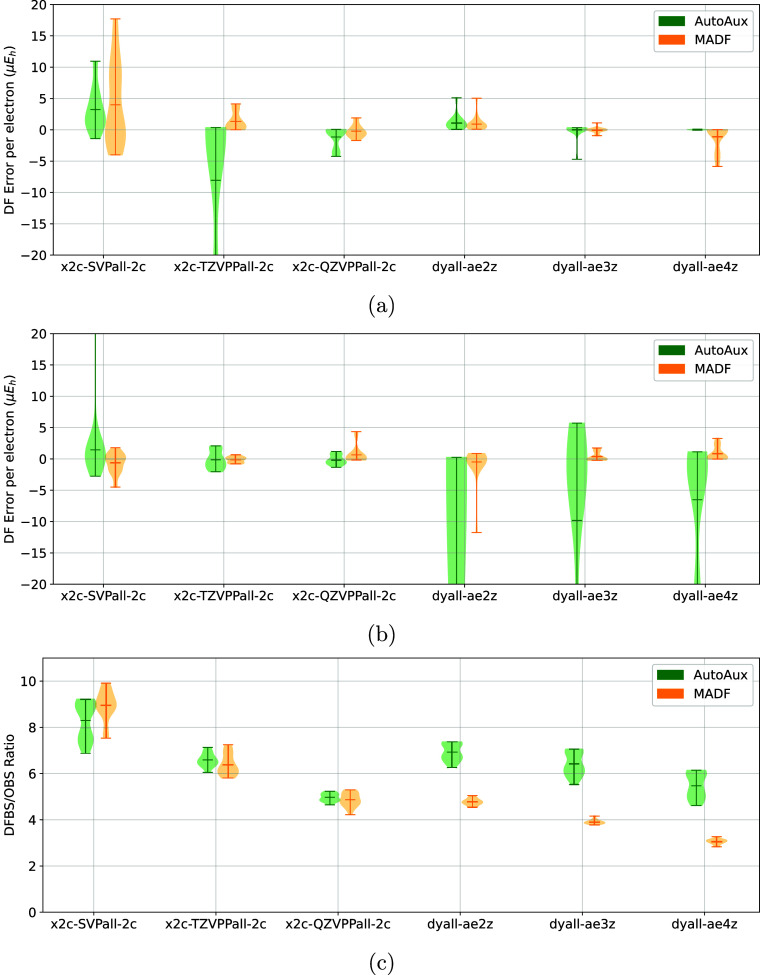
Comparison of DF errors of (a) X2C-HF and (b) X2C-MP2 energies of the Tm60 test set obtained with AutoAux and MADF DFBSs. (c) Comparison of DFBS sizes, relative to that of the corresponding OBS.

**Figure 11:**
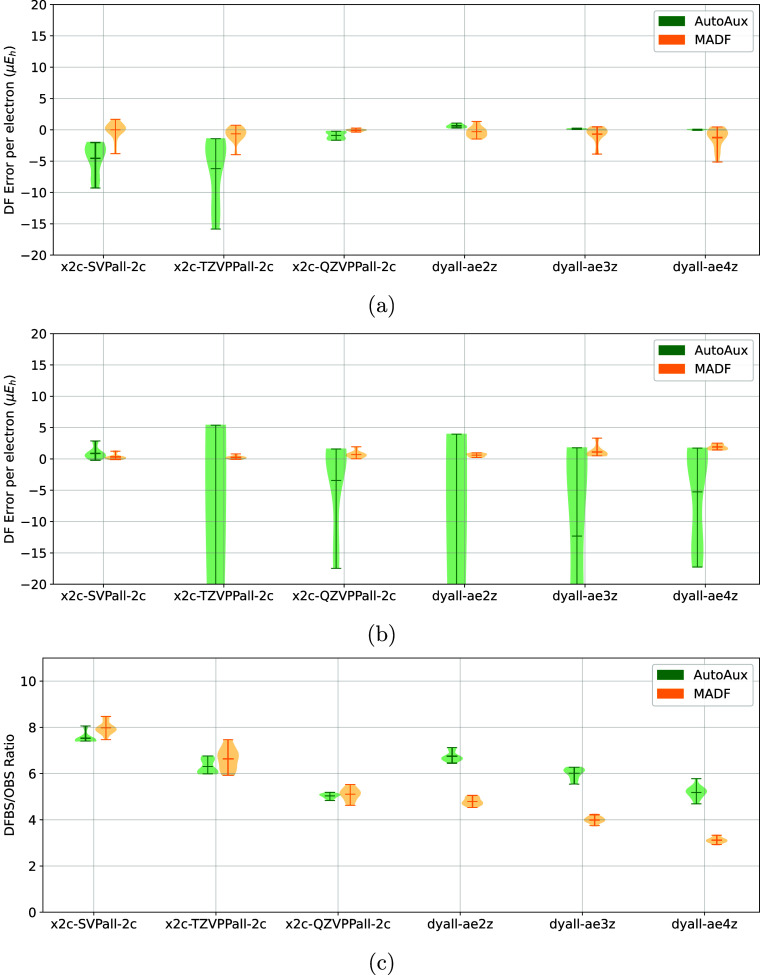
Comparison of DF errors of (a) X2C-HF and (b) X2C-MP2 energies of the Ln54 test set obtained with AutoAux and MADF DFBSs. (c) Comparison of DFBS sizes, relative to that of the corresponding OBS.

The AutoAux exponent ranges are significantly reduced relative to that of Lehtola’s pCD-derived DFBS, and the exponents are well separated owing to even-tempering. However, it is clear that the optimal DFBS exponents are not necessarily even-tempered, as the exponent density of the complete and pCD-processed DFBS of Lehtola and that of the manually optimized DFBS has significant nonuniformity. Ideally, one would also be able to avoid varying the exponent ratios with *L* for the even-tempering recipe.

In this work, we regularize the complete candidate exponent pool using a simple adaptive algorithm designed to produce noneven-tempered sets that deviate from the input set as little as possible and with exponent ratios as least as large as the target ratio threshold. Given a primitive SHG set C and a target exponent ratio threshold ζ, each subset $C_{L}$ containing primitives of angular momenta *L* is regularized by repeatedly replacing the pair of primitive SHG shells with the smallest exponent ratio α_1_/α_2_ by a single primitive SHG shell with the geometric mean exponent $\alpha =\sqrt{\alpha_{1}\alpha_{2}}$ . If multiple exponent pairs have a “soft” tie for the smallest exponent ratio, the pair with the smallest orbital exponent wins. Regularization stops if no pairs of primitives have an exponent ratio less than ζ. Clearly, regularization is most easily implemented if $C_{L}$ is first sorted by the exponent in descending order. The algorithm is described in detail in Algorithm 1. The recommended value of ζ will be determined heuristically in Section 4.1.
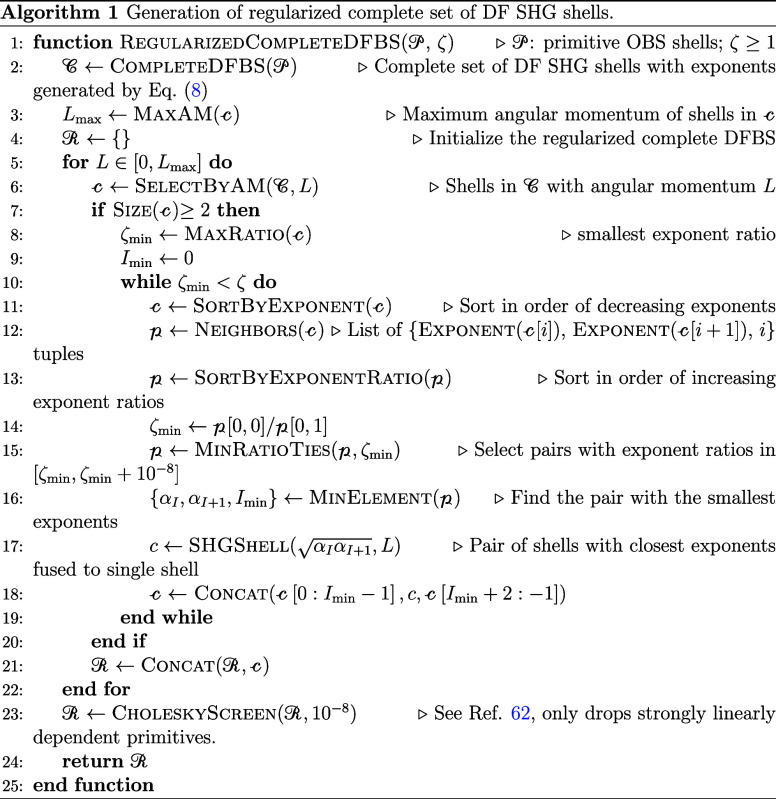


### 2.3 Estimating Energy Contribution of DFBS Primitives

In a single atom, DFBS must contain solid harmonics with *L* up to 2*L*_OBS_ to obtain exact values for all possible matrix elements of the Hamiltonian in OBS, and the above regularization methods do not change this fact. However, it is well-known that the importance of DF AOs with the highest angular momenta is relatively low, at least for energies. Manually optimized DFBSs typically restrict the *L*_DFBS_ to significantly below 2*L*_OBS_, since only DFBS with *L* up to *L*_OBS_ + *L*_occ_ are needed for exact representation of (*ov*|*ov*) integrals needed for the MP2 energy. In practice MP2-targeted DFBS are used successfully in the context of higher-order methods, like coupled-cluster. In ad hoc DFBS generators like AutoAux, the angular momenta of DFBS are restricted heuristically, i.e., *L*_max,DF_ = max­(2*L*_occ_, *L*_OBS_ + *L*_inc_), where *L*_inc_ is fixed to 1 or 2 depending on *Z*.^61^ To avoid the heuristics as much as possible, we attempted to design a first-principles energy-based model for further pruning of DFBS.

Consider the expectation value of a 2-body Fock-space Hamiltonian:

$$E=\sum_{pq}h_{p}^{q}\gamma_{q}^{p}+\frac{1}{2}\sum_{pqrs}h_{pq}^{rs}\gamma_{rs}^{pq}$$ 9

where *h*_*p*_^*q*^ and *h*_*pq*_^*rs*^ are the Hamiltonian matrix elements and γ_*q*_^*p*^ and γ_*rs*_^*pq*^ are the elements of the 1- and 2-body reduced density matrices, respectively. Since DF is used to approximate the Coulomb integrals *h*_*pq*_^*rs*^ ≡ (*pr*|*qs*) ≡ (*pr*|*Ĵ*|*qs*), we will focus on the two-body part of the energy:

E2=12∑pqrs(pr|qs)γrspq 10

Our objective is to quantitatively estimate the energetic contribution of a given AO in a given DFBS to ^2^ *E*, using a first-principles model for the 2-RDM. The most straightforward approach would be to evaluate ^2^ *E* with the Coulomb integral approximated via robust DF,

$$\left(pr|qs)=\sum_{XY}(pr|X)(J^{-1}{)}_{XY}(Y|qs\right)$$ 11

where (*pq*|*X*) ≡ (*pq*|*Ĵ*|*X*) and (**J**)_*XY*_ ≡ (*X*|*Ĵ*|*Y*). The magnitude of the change in DF-approximated ^2^ *E* due to the inclusion of the candidate DF AO in DFBS could be used to gauge its importance for accurate computation of ^2^ *E*. There are two problems with such an approach. positive (electrostatic) and negative (exchange, correlation) contributions. Thus, contributions from a given DF AO to the positive and negative parts of ^2^ *E* could cancel each other spuriously, causing an artificial omission of such AO from DFBS. Replacing the summands in eq 10 by their magnitudes would resolve the sign issue but would, in effect, change the weights of different components of the energy. Second, it is known that the charge density fitting used to compute the electrostatic energy only requires fitting functions with lower angular momenta; thus, it makes sense to tune the fitting basis to the more challenging exchange and correlation contributions to the energy. Therefore, we model the energetic importance of DF AOs by the diagonal exchange-like contribution to ^2^ *E* obtained from eq 10 by substitution *rs* → *qp*:

Edx2≡12∑pq(pq|qp)γqppq=−12∑pq(pq|qp)γpqpq 12

Note that γ_*pq*_^*pq*^ ≥ 0, hence each summand in eq 12 is positive (real orbitals are assumed for brevity).

Another question is how to obtain the 2-RDM. Our original efforts to model the importance of DFBS focused on heuristic models of RDMs (such as the Fermi–Dirac distribution). Thus, in practice we constructed an estimator that requires a model for *orbital occupancies n*_*p*_ ≡ γ_*p*_^*p*^ only, rather than the full 2-RDM. To model the 2-RDM in terms of orbital occupancies, we use the Cauchy-Schwarz inequalities for the 2-RDM,^83^ which for positive γ_*pq*_^*pq*^ becomes simply:

$$\gamma_{pq}^{pq}\leq \sqrt{n_{p}n_{q}}$$ 13

This leads to the following bound for ^2^ *E* ^dx^ in terms of orbital occupancies:

|Edx2|≡−Edx2≤12∑pq(pq|qp)npnq≡E®dx2 14

This expression is independent of whether the single-particle states are spin-free or spin–orbital. Since robust DF guarantees that the error in (*pq*|*qp*) = (*pq*|*pq*) is positive:

$$(pq|pq)-(\tilde{pq}|\tilde{pq})\overset{\mathrm{robust}\;\mathrm{DF}}{\geq }0$$ 15

then the robust DF approximation to eq 14

E̅~dx2≡12∑XY∑pq(pq|X)(J−1)XY(Y|pq)npnq 16

is guaranteed to be smaller than the exact value. This gives us the ability to estimate the importance of a given DF AO for modeling ^2^ *E̅* ^dx^ by monitoring its effect on E̅~dx2 as well as the ability to control the overall error in ^2^ *E̅* ^dx^.

The angular momentum conservation ensures that *X* and *Y* in eq 16 must have the same angular momentum. This makes it convenient to split E̅~dx2 into its angular momentum components:

E̅~Ldx2≡12∑XYlX=lY=L∑pq(pq|X)(J−1)XY(Y|pq)npnq 17

where *l*_*X*_ is the angular momentum of DF AO *X*. Practical tests showed that it is important to represent E̅~Ldx2 for *L* ≤ 2*L*_occ_ more accurately than the higher *L* channels. Practical tests also showed that E̅~Ldx2 grows with *Z*, hence it makes sense to prune DFBS according to the value of E̅~Ldx2 per unit nuclear charge.

The next question is in which order to consider the candidate DF AOs. To decide the order we rewrite the E̅~Ldx2 in terms of contributions from individual DF AOs:

ϵ̅~Xdx2≡12∑YlY=lX∑pq(pq|X)(J−1)XY(Y|pq)npnq 18

E̅~Ldx2=∑XlX=Lϵ̅~Xdx2 19

Although ^2^ϵ̅_*X*_^dx^ are not strictly positive, they are nearly so; all negative values are very small (on the order of tens of microhartrees). For each angular momentum channel *L* vector |^2^ϵ̅_*X*_^dx^| is computed once and sorted in descending order; this defines the order in which DF AOs are considered.

2-body energy pruning of the candidate DF AO pool $R^{L}$ produced by Algorithm 1 proceeds as follows. If E̅~Ldx2 from (eq 17) is less than *Z*τ, where τ is the target threshold, then all DF AOs of this angular momentum are discarded, else the DF AOs are sorted according to the order of importance provided by |^2^ϵ̅_*X*_^dx^|. The candidate DF AOs are added to the *L*-channel of pruned DFBS, $D^{L}$ , until the difference between E̅~Ldx2 evaluated with $R^{L}$ and with $D^{L}$ is smaller than *Z*τ. For an atom with ground-state configuration including occupied subshells of angular momenta ≤*L*_occ_ DF channels *L* ≤ 2*L*_occ_ are pruned with τ = τ_1_, the rest are pruned with τ = τ_2_. For atoms with *L*_occ_ = 0, namely H, He, Li, and Be, to avoid overpruning of the higher *L* DF channels, we consider *L*_occ_ to be 1. The values of τ_1_ and τ_2_will be determined heuristically in Section 4.1.

### 2.4 Correlated Model of Orbital Occupancies

Our initial instinct was to model orbital occupancies in eq 14 by Fermi–Dirac distribution (with heuristics for the temperature). Unfortunately such an approach did not prove fruitful. Using atomic mean-field ensemble orbitals often used as initial guess for atomic density matrix would not suffice, since this will fail to generate nonzero populations for orbitals important in dynamical electron correlation. Therefore, we developed a simple model for correlated 1-RDM of a neutral atom in an ensemble state using spin-opposite scaled (SOS) first-order Møller–Plesset perturbation of a model mean-field ensemble density. The mean-field density in OBS is obtained by one-shot diagonalization of OBS Fock matrix **F** computed from the atomic ensemble density generated by populating subshells of a minimal basis using the atom’s reference ground-state electron configuration. Such definition for the OBS Fock matrix is utilized in the Libint library and the MPQC program under the name “superposition of atomic densities” (SOAD) for generating guess Fock matrices for SCF. SOAD can be viewed as a simplified version of the popular SAD method,^87^ with differences described for completeness in Appendix A.

Diagonalization of SOAD ensemble Fock matrix expressed in OBS AO (see Appendix A)­

$$F_{\mathrm{SOAD}}C=\mathrm{SC}\varepsilon$$ 20

produces energies and OBS AO coefficients of nonself-consistent ensemble orbitals. In this work, we have used the spin-free exact two-component 1-body (core) Hamiltonian (sf-1eX2C)^88–90^ to construct the **F**_SOAD_ matrix to account for scalar relativistic effects. In general the model orbital energies of the atomic subshells obtained from eq 20 do not match the canonical order of Aufbau principle (Madelung rule) or even the order produced by self-consistent field in the given OBS. Thus, the uncorrelated occupation numbers for the OBS ensemble orbitals **n** ^(0)^ are obtained by mapping the spherically averaged MBS ensemble occupancies **n**_SOAD_ (Appendix A) via:

$$n^{\left(0\right)}=\mathrm{id}(C^{†}S_{\mathrm{OBS},\mathrm{MBS}})n_{\mathrm{SOAD}}$$ 21

where **S**_OBS,MBS_ is the overlap of OBS and MBS AOs, and id maps the real-valued matrix argument to the nearest identity matrix. Columns in **C** corresponding to zero and nonzero ensemble occupancies are deemed unoccupied (*a*, *b*) and occupied (*i*, *j*), respectively. These orbitals and the corresponding orbital energies are used to compute opposite-spin ensemble Møller–Plesset first-order amplitudes:

$$t_{ab}^{ij}=-\frac{\sqrt{n_{i}^{\left(0\right)}n_{j}^{\left(0\right)}}}{2}\frac{\left(ia|jb\right)}{\varepsilon_{a}+\varepsilon_{b}-\varepsilon_{i}-\varepsilon_{j}}{\left(1-e^{-\kappa (\varepsilon_{a}+\varepsilon_{b}-\varepsilon_{i}-\varepsilon_{j})}\right)}^{2}$$ 22

The last factor on the right-hand side is the κ-regularizer used by Lee and Head-Gordon^91^ in the context of orbital-optimized MP2 method. Whereas in ref 91. heuristic tuning suggested κ = 1.45 *E*_h_^–1^ as best at avoiding the singularities and accounting for the inaccuracy of the MP1 amplitude model. Here we set κ = 3 *E*_h_^–1^ as the purpose of the regularizer here is to only avoid potential singularities due to possible degeneracies between occupied and unoccupied subshells; the inadequacy of the MP1 model for describing correlations of electrons in the partially occupied subshells is already partially addressed by avoiding internal excitations within such subshells. The corresponding second-order correlation contributions **n** ^(2)^ to orbital occupancies are obtained straightforwardly:

$$n_{i}^{\left(2\right)}=\sum_{jab}-2t_{ab}^{ij}t_{ij}^{ab}$$ 23

$$n_{a}^{\left(2\right)}=\sum_{ijb}2t_{ab}^{ij}t_{ij}^{ab}$$ 24

The total correlated orbital occupancy vector **n** is a sum of the mean-field and correlated contributions:

$$n=n^{\left(0\right)}+n^{\left(2\right)}$$ 25

### 2.5 The MADF Algorithm

For a given (input) OBS consisting of contracted SHG AOs on an atom with atomic number *Z* MADF (1) generates a “complete” set of primitive DF SHG AOs necessary to represent all products of uncontracted OBS AOs using the method of Lehtola,^62^ (2) regularizes it by fusing pairs of shells with overly close exponents as described in Section 2.2, and (3) prunes out DF AOs not important for description of correlated 2-body energy using the method described in Section 2.3. The complete description of the algorithm is given in Algorithm 2. Its 3 model parameters (with 4 unique values) are summarized in Table 1.
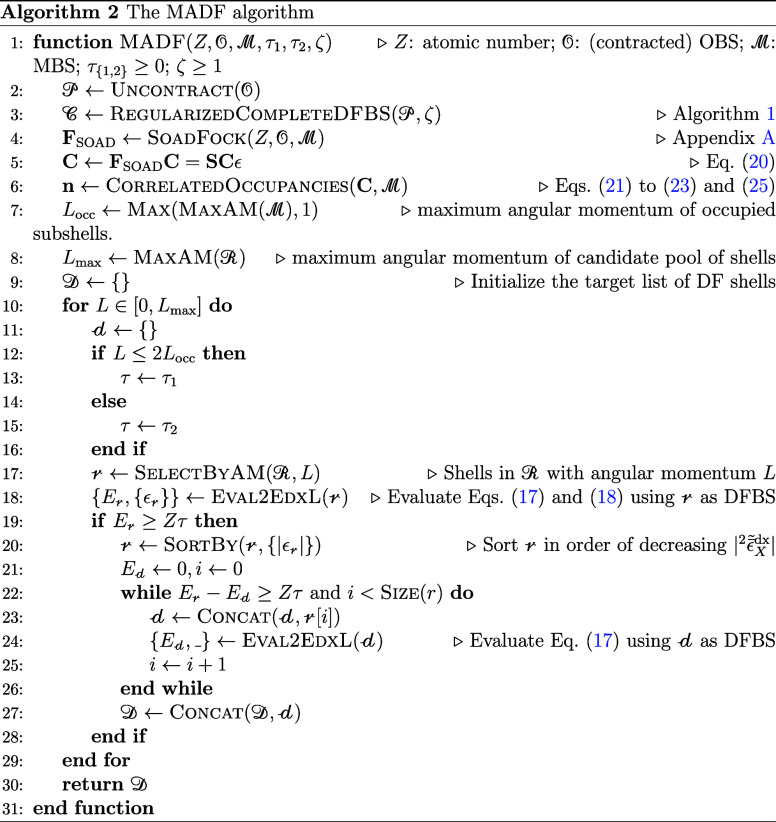


**Table 1:** Model Parameters of the MADF DFBS Generator

parameter	description	values
ζ	exponent ratio threshold for Algorithm 1	1.4
τ_1_	2-body energy threshold for *L* ≤ 2*l* ^occ^	10^–6^ (nr), 10^–7^ (rel)^a^
τ_2_	2-body energy screening threshold for *L* > 2*l* ^occ^	10^–5^

a Optimal values of τ_1_ are different for nonrelativistic and relativistic OBSs, see Sections 4.1.1 and 4.1.2.

## 3 Technical Details

The MADF algorithm is implemented in a developmental version of MPQC;^92^ generation of base DFBS by Lehtola’s algorithm^62^ was implemented in Libint.^93,94^ For optimization of MADF parameters and its benchmark testing, we employed the nonrelativistic and relativistic Hartree–Fock (HF) and second-order Møller–Plesset (MP2) methods. DF errors were computed relative to the HF and MP2 energies obtained with exact (four-center) two-electron matrix elements throughout. The exact two-component decoupled one-body Hamiltonian^88–90^ (1eX2C, or simply X2C) with spin–orbit coupling was used for all relativistic computations without empirical scaling corrections. The core orbitals were frozen in nonrelativistic MP2 computations with cc-pVXZ and def2-OBSs.

The MADF model parameters for use with nonrelativistic OBS were determined on training set TS1 composed of 38 closed-shell systems with light elements: AlF_3_, Ar, BF_3_, BH_3_, C_2_H_2_, C_2_H_4_, C_2_H_6_, CH_3_OH, CH_4_, Cl_2_, CO_2_, COH_2_, CS_2_, F_2_, H_2_O, HCl, HF, HNO_2_, N_2_, cis-N_2_H_2_, trans-N_2_H_2_, Ne, NH_3_, NOCl, O_2_, O_3_, OF_2_, P_2_, PF_3_, PH_3_, S_2_, SF_2_, SiH_4_, SiO_2_, and SO_2_. The performance of the recommended model parameters was assessed on the standard G2 set of molecules,^95^ containing 118 closed- and 30 open-shell systems. For training and assessment computations cc-pVXZ,^38^ cc-pwCVXZ^40^ (X = D, T, Q) and def2-SVP, def2-XZVP (X = T, Q)^44^ were used. Since there are no cc-pwCVXZ basis sets for Li, Be, and Na atoms, systems containing these atoms were skipped from the assessment of these OBSs.

The MADF model parameters for use with relativistic OBS were determined on training set TS2 composed of 24 closed-shell systems with heavy elements: AgH, AsH_3_, AuH, BiH_3_, CuH, GaH_3_, GeH_4_, HAt, HBr, Hi, InH_3_, Kr, PbH_4_, PoH_2_, PtC, RuC, SbH_3_, ScH, SeH_2_, SnH_4_, TeH_2_, TlH_3_, Xe, and Rn. The performance of the recommended model parameters was assessed on 2 sets: (a) the Ln54 benchmark set composed of 54 rare-earth compounds,^96^ and (b) the set of 60 diatomics containing d-block (transition metal) atoms from ref 97 (we will refer to this set as Tm60). Due to the challenging SCF orbital optimization landscape and to make the assessment as robust and automated, for each OBS we did assessment on the Ln54 and Tm60 systems for which the exact (four-center) X2C-HF SCF converged in 30 or fewer SCF iterations. The training and assessment was performed with the following OBSs designed for spin–orbit relativity: X2C-SVPall-2c,^52^ X2C-XZVPPall-2c^53^ (X = T, Q), and all-electron Dyall basis sets dyall-ae*Y*z^54–56^ (*Y* = 2, 3, 4).

The performance of MADF was assessed also against the AutoAux DFBS generator^61^ as implemented in BSE’s command-line interface.^41^ For cc-pVXZ, cc-pwCVXZ, and def2-family of OBSs we also compared against the corresponding manually optimized DFBS cc-pVXZ-RIFIT, cc-pwCVXZ-RIFIT (X = D, T, Q) and def2-SVP-RIFIT and def2-XZVP-RIFIT (X = T, Q) for the G2 set for comparison. acCD calculations were performed using OpenMolcas version 24.10.^98^ Since this version of OpenMolcas does not have an implementation of open-shell MP2, for comparison of MADF and acCD only the closed-shell subset of G2 set was used. We also show acCD vs MADF comparison only for the cc-pVXZ family of basis sets. All geometries, except for the G2 set, were optimized with the PBE0 ^99^ and def2-TZVP basis set using the Psi4’s^100^ geometry optimization module. For elements with atomic number greater than 36, the effective core potentials (ECPs)^101^ available for def2-TZVP were used for geometry optimization.

All matrix pseudoinverses and pseudoinverse square roots were computed with Löwdin’s orthogonalization procedure.^102^

## 4 Results

### 4.1 Training MADF Model Parameters

The MADF model parameters were tuned to ensure that that the DF errors for the respective training sets did not exceed target accuracies of ±20μ*E*_h_ and ±10μ*E*_h_ per electron for HF and MP2 energies, respectively. This target range for errors was determined by analyzing the DF errors of manually optimized DFBSs; the majority have larger errors than these targets (see Section 4.2.1).

#### 4.1.1 Training τ_1,2_ and ζ for Nonrelativistic OBS

The variation of DF errors with τ_2_ was studied next, by keeping the rest of the parameters fixed (τ_1_ = 10^–8^, ζ = 1.2) sufficiently closely to their asymptotic limits (τ → 0, ζ → 1) without causing ill-conditioning and excessive costs. As Figure 2a,b indicate, the DF errors of HF and MP2 are largely converged at τ_2_ = 10^–5^. Next, τ_2_ was fixed at 10^–5^ and τ_1_ was varied (Figure 3a,b). The DF errors are sufficiently converged with τ_1_ = 10^–6^. Finally, with τ_1_ = 10^–6^ and τ_2_ = 10^–5^ variation of DF errors with ζ was studied (Figure 4a,b). For ζ > 1.4 the max DF errors increase due to the insufficient spanning of the OBS AO product space by the DFBS AOs, whereas for ζ < 1.2 (not shown) the onset of ill-conditioning in DFBS makes the MADF model not numerically stable. ζ = 1.4 ensures sufficient convergence of the DF errors without undue numerical problems.

Note that ζ = 1.4 is significantly smaller than the exponent ratio 1.8 used by the AutoAux generator (1.8)^61^ and ratio 2 used by PySCF’s generator^67^ for even-tempered DFBS construction. Our findings confirm that the relatively small exponent ratios are indeed necessary for accurate density fitting. For example, exponent ratios in the [1.3, 1.5] range are quite common in the *L* ≤ 2 channels of the manually optimized def2-QZVPP-RIFIT DFBS (see Figure 1). Clearly even-tempered spanning of the AO product space is suboptimal for practical basis sets.

#### 4.1.2 Retraining MADF Parameters for Relativistic OBS

Parameters τ_1,2_ and ζ were trained again using the relativistic HF and MP2 energies of the TS2 set. τ_2_ was varied first, with τ_1_ = 10^–8^ and ζ = 1.3. Here we start with ζ = 1.3 instead of ζ = 1.2 like in the nonrelativistic case because ζ = 1.2 is too small and causes numerical issues with the relativistic OBSs (Figure 5a,b); just as in nonrelativistic computations τ_2_ = 10^–5^ ensures sufficient convergence of the DF errors. τ_1_ was then varied with fixed τ_2_ = 10^–5^ and ζ = 1.3 (Figure 6a,b). In contrast to the nonrelativistic case, a much smaller τ_1_ is needed for sufficient convergence of the DF errors. The variation of relativistic DF errors with ζ (Figure 7a,b) is similar to the nonrelativistic case, with ζ = 1.4 deemed sufficient. Yet again, this is a much smaller ratio than the ratios used by even-tempered DFBS generators.^61,67^

The optimal values of the parameters MADF are briefly tabulated in Table 1.

### 4.2 Assessment of DFBSs Generated by the MADF Generator

#### 4.2.1 The G2 Set

The DF errors of HF and MP2 energies of the G2 test set produced with DFBS generated by the MADF generator with the recommended model parameters were compared against those with manually optimized DFBS as well as against those generated by the AutoAux generator. The MADF DFBSs produce smaller DF errors in HF energies than the manually optimized DFBS (Figure 8a), since manually optimized DFBSs are solely optimized to reduce DF-/RI-MP2 errors. However, the MADF DFBSs also outperform the manually optimized counterparts for MP2 energies (Figure 8b). However, the manually optimized DFBS are significantly more compact (by as much as a factor of 2) than the MADF counterparts, although the gap decreases significantly for quadruple-ζ basis sets (Figure 8c). The comparison to the AutoAux generator is more interesting. Despite fewer parameters, MADF DFBSs produce similar DF errors in HF and MP2 energies. This is especially striking since the MADF DFBS are more compact than the AutoAux counterparts, sometimes by a significant margin (such as for def2-TZVP).

We have also compared the performance of primitive DFBSs produced by MADF with contracted DFBSs produced by acCD. From Figure 9a it is clear that MADF DFBSs produce smaller DF errors in HF energies compared to acCD DFBSs. As can be seen in Figure 9b the MP2 errors for cc-pVDZ are smaller with MADF compared to acCD and for cc-pVTZ and cc-pVQZ the magnitude MP2 errors are <5μ*E*_h_ per electron, which is sufficient. MADF also provides significantly smaller DFBSs than that of acCD with virtually similar errors for cc-pVTZ and cc-pVQZ as seen in Figure 9c.

#### 4.2.2 Ln54 and Tm60 Sets

The value of DFBS generators is especially pronounced for heavier elements where manually optimized DFBS are simply not available. Figures 10 and 11 illustrate the relative performance of MADF and AutoAux DFBSs for relativistic computations on molecules with d-block and f-block elements, respectively. It is evident from Figures 10a and 11a that the DF errors of the X2C-HF energies are smaller with MADF DFBSs than with AutoAux DFBSs, especially for the TURBOMOLE sets. For the MP2 energies (Figures 10b and 11b) MADF DFBSs are also more accurate, especially for the Dyall basis sets. Although for the TURBOMOLE OBSs MADF DFBS are slightly larger than the AutoAux counterparts, the smaller errors (especially for the triple-ζ OBS) make the increase in the basis set palatable. For the Dyall basis sets MADF generated significantly smaller DFBSs than AutoAux, while producing comparable DF errors of HF energies and much smaller DF errors of MP2 energies.

## 5 Summary

This work introduced the MADF algorithm for generating density-fitting basis set composed of primitive solid-harmonic Gaussian AOs and suited for nonrelativistic and relativistic (all-electron) computations with mean-field and correlated electronic structure models. Not using even-tempered AO sets for spanning the AO product space, as in other comparable generators,^61,67^ produces compact and accurate candidate pools of DFBS AOs while sufficiently reducing the numerical redundancy. The subsequent pruning of the candidate DFBS pool based on the 2-body energy as the importance metric allows to naturally reduce the relevant range of angular momentum channels and exponent spans with minimal use of heuristics. Generation of primitive (rather than contracted) DF AOs allows to keep the integral evaluation costs optimally low. Using only 3 model parameters MADF generates basis sets that match or exceed the accuracy-to-size ratio of the state-of-the-art DFBS generators; the comparison is particularly favorable with OBSs suited for relativistic all-electron computations. Computations utilizing MADF DFBS, of course, can also benefit from system-specific DFBS compressions.^69,103^

Although picture change and other relativistic effects on the 2-particle interaction were not considered here, it will be interesting to examine the impact of such effects on the optimal MADF model parameters in the future; it is known that there are nontrivial differences in the requirements on the DFBS that stem from relativistic contributions to the effective 2-particle interactions.^13^ Even in the nonrelativistic framework much more testing of the approach will be clearly needed. Most important is to test the performance in the context of higher-order correlated methods (especially coupled cluster), for DF approximation of non-Coulomb operators, for properties other than energy, and for other basis set families (e.g., multiply augmented correlation-consistent sets, ANO, etc.). Early experiences with MADF DFBSs appear promising. For example, we recently utilized the MADF DFBS generator for explicitly correlated (F12) coupled-cluster computations with aug-cc-pV7Z OBS (for which no manually optimized DFBS is available) where we found that the DF errors with the MADF DFBS are significantly smaller than the use of the manually optimized aug-cc-pV6Z-RIFIT basis.^104^ These and other experiences with MADF DFBSs will be reported elsewhere.

## Supplementary Material



## A Superposition of atomic densities (SOAD)

Unlike the SAD method,^87^ with computes the Fock matrix from the atomic (optionally, spin-free) ensemble density matrix computed in OBS self-consistently, SOAD Fock matrix is computed from fixed spin-free ensemble occupancies **n**_SOAD_ of the minimal basis set (MBS) AOs, without self-consistency. **n**_SOAD_ for a single atom in an orthonormal MBS is obtained by smearing each subshell’s electrons evenly among all of its orbitals; e.g., for a carbon atom occupancies of 1s, 2s, 2p_*x*_, 2p_*y*_, and 2p_*z*_ MBS AOs are 2, 2, 2/3, 2/3, and 2/3, respectively. This recipe has been referred to as average-of-configuration (AOC)^105^ and canonical ensemble.^106^ The number of electrons in each subshell is defined by the electron configuration of that atom’s neutral ground state taken from NIST Atomic Spectra Database.^107^ For a molecule **n**_SOAD_ is obtained by concatenation of the atomic occupancies, without taking into account the effects of nonunit molecular MBS metric. In this work the MINI basis^108,109^ was used as MBS for *Z* ≤ 54, and the ANO-RCC-MB basis^110^ for 54 < *Z* ≤ 96. **n**_SOAD_ is used to construct SOAD Fock matrix in OBS AO basis, **F**_**SOAD**SOAD_, as follows:

$$\left(F_{\mathrm{SOAD}}{)}_{\nu }^{\mu }=h_{\nu }^{\mu }+\sum_{m}(n_{\mathrm{SOAD}}{)}_{m}[2(\mu \nu |mm)-(\mu m|\nu m)\right]$$
26

where *h*_ν_^μ^ are the matrix elements of the 1-body (core) Hamiltonian (sf-1eX2C^88–90^ in this work), *m* runs over MBS AOs and μ, ν over OBS AOs. It should be noted that eq 26 is similar to the projection-free guess proposed by Norman and Jensen^111^ for Dirac-Hartree–Fock/Kohn–Sham.

## Notes

MADF density-fitting basis sets generated in this work in the Gaussian 94 basis set format; molecular geometries of all species (PDF)
